# Quantifying Spike Train Oscillations: Biases, Distortions and Solutions

**DOI:** 10.1371/journal.pcbi.1004252

**Published:** 2015-04-24

**Authors:** Ayala Matzner, Izhar Bar-Gad

**Affiliations:** The Leslie & Susan Goldschmied (Gonda) Multidisciplinary Brain Research Center, Bar-Ilan University, Ramat-Gan, Israel; Philipps-University Marburg, GERMANY

## Abstract

Estimation of the power spectrum is a common method for identifying oscillatory changes in neuronal activity. However, the stochastic nature of neuronal activity leads to severe biases in the estimation of these oscillations in single unit spike trains. Different biological and experimental factors cause the spike train to differentially reflect its underlying oscillatory rate function. We analyzed the effect of factors, such as the mean firing rate and the recording duration, on the detectability of oscillations and their significance, and tested these theoretical results on experimental data recorded in Parkinsonian non-human primates. The effect of these factors is dramatic, such that in some conditions, the detection of existing oscillations is impossible. Moreover, these biases impede the comparison of oscillations across brain regions, neuronal types, behavioral states and separate recordings with different underlying parameters, and lead inevitably to a gross misinterpretation of experimental results. We introduce a novel objective measure, the "modulation index", which overcomes these biases, and enables reliable detection of oscillations from spike trains and a direct estimation of the oscillation magnitude. The modulation index detects a high percentage of oscillations over a wide range of parameters, compared to classical spectral analysis methods, and enables an unbiased comparison between spike trains recorded from different neurons and using different experimental protocols.

## Author Summary

Neuronal oscillations play a key role in normal behavior and during multiple pathological conditions. In this manuscript, we expose major biases and distortions which arise from the quantification of neuronal spike train oscillations. These, previously neglected, biases hinder the comparison of oscillations across brain regions, neuronal types and behavioral states, leading inevitably to severe misinterpretation of experimental results. We demonstrate the biases computationally, formulate them analytically and validate their appearance and magnitude in an experimental dataset recorded from Parkinsonian non-human primates. Next, following a formulation of the distortions, we introduce a novel objective measure, the "modulation index", which overcomes these biases, and enables a reliable detection of oscillations from spike trains and a direct estimation of the oscillation magnitude. The modulation index is validated on the same experimental data demonstrating the unbiased detection of beta oscillation in the globus pallidus during Parkinsonism. The manuscript provides a solid infrastructure for oscillation analysis which benefits multiple neuroscience fields ranging from basic science to clinical studies, moreover its results may be expanded to encompass additional fields in biology which require the spectral analysis of point process data.

This is a *PLOS Computational Biology* Methods paper

## Introduction

Neuronal oscillations play a key role in normal behavior and in different pathological conditions [1–3]. Neuronal oscillations are typically classified into a range of frequencies including the delta (1–4Hz), theta (4–8Hz), alpha (8–12Hz), beta (12–30 Hz), and gamma (30–80Hz) bands [3]. Enhanced expression of specific oscillatory frequencies or oscillations in a broader band is perceived as indicative of different normal functions, such as the enhanced gamma preceding movement [1] and different pathological conditions such as the enhanced beta associated with Parkinsonism [4]. Analysis of the power spectrum is a common method for identifying enhanced (or reduced) oscillations in neuronal data, and is widely used on a variety of brain signals spanning multiple orders of magnitude, such as electroencephalograms (EEG), local field potentials (LFP), multiunit activity (MUA), single unit spike trains and cellular membrane potentials [5–8].

The time of occurrence of action potentials emitted by a single neuron; i.e., single unit spike trains, are a major source of neurophysiological data stemming from both intracellular and extracellular recordings. These neuronal spike trains may be viewed as a stochastic point process where a discrete event represents each action potential [9]. The generation of each spike within the train is assumed to be dependent on an underlying instantaneous firing rate. The resultant point process reflects the originating rhythm only partially since the individual events are stochastically generated from the rate function [10]. Thus, despite an underlying oscillatory firing rate, in most cases the neuron will skip a large portion of the oscillation cycle or even entire cycles [11]. The most simplistic statistical spike train model assumes that the generation of each spike is dependent solely on the underlying instantaneous firing rate, and is independent of all other previous spikes. This model is termed the inhomogeneous Poisson process when the instantaneous firing rate changes over time. Spectral analysis of an experimentally recorded spike train under this assumption is thus assumed to reflect the oscillations of the underlying instantaneous rate directly. However, different properties of the neuron or the experiment can cause the spike train to reflect its underlying oscillatory firing rate well or poorly, and hence can facilitate or impede the detection of an underlying oscillation [7,10].

In this paper, we address the problem of oscillation detection in spike trains. First, we quantify the influence of different biological and experimental factors on the detection of the spectral peak and its significance. We then analytically derive a solution for the modulation index of an oscillatory Poissonian neuron and present a novel objective measure that enables reliable detection of oscillations in spike trains. We investigate the oscillations in experimentally recorded neurons from Parkinsonian primates, and compare these experimental results to our numerical and analytic findings. Finally, we derive a solution for the evaluation of the actual recording duration required for the detection of spike train oscillations in experimental data.

## Results

**Fig 1:**
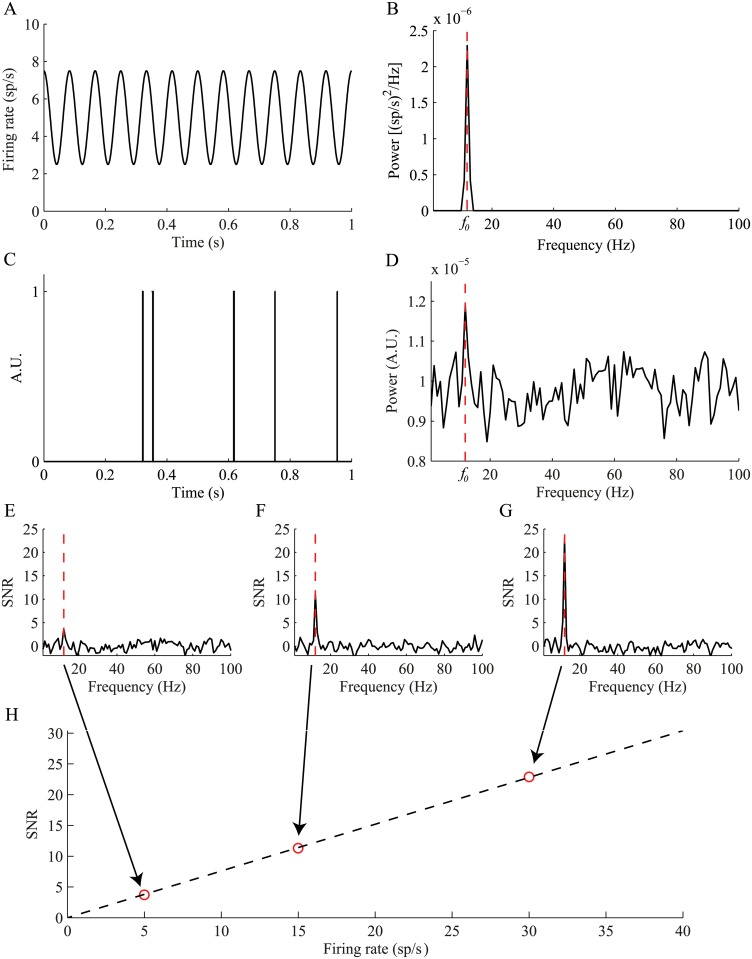
Limited representation of oscillations following a rate to spike train transformation. (A) A short (1 second) segment taken from an oscillatory rate function (*f*_0_ = 12 Hz, *r*_0_ = 5 sp/s and *m* = 0.5). (B) Power spectrum of a 5 minute segment taken from the rate function. The dashed vertical line indicates *f_0_*. (C) A short (1 second) example of a Poisson generated spike train from the same rate function. (D) Power spectrum of the 5 minute spike train. (E-G) The power spectrum normalized to SNR units, when the base firing rate is (E) 5 sp/s, (F) 15 sp/s, and (G) 30 sp/s. (H) The peak oscillation amplitude as a function of the mean firing rates. The circles indicate the *f_0_* oscillations shown in examples E-G, and the solid line indicates the fitted linear function.

The spiking activity of oscillatory neurons can be modeled as an inhomogeneous Poisson process whose rate λ(*t*) is modulated by a cosine function (Fig 1A):

$$\lambda (t)=r_{0}\cdot (1+m\cdot \text{cos}(2\pi \cdot f_{0}\cdot t))$$ (1)

where *r*_0_ is the baseline firing rate, 0 ≤ *m* ≤ 1 is the modulation index, and *f*_0_ is the oscillation frequency. Classically, the oscillatory nature of the neuronal activity is assessed using spectral estimation methods. The power spectrum of this rate function enables the identification of the oscillation base frequency (*f*_0_) which appears as a clear peak in that frequency, while all the other frequencies have no power (Fig 1B). However, estimation of the power spectrum of the Poisson generated spike train, ρ(t), from this rate function (Fig 1C) results in a reduced peak at the base frequency and increased power in all the other frequencies, such that detection of the base frequency is not straightforward (Fig 1D). The power spectrum can be normalized to reflect the signal—to—noise ratio (SNR) in standard deviations of the power in each frequency relative to the mean power in the 100–500 Hz frequency band of the spike train which serves as a noise baseline for different values of the baseline rate (Fig 1E–1G). The power spectrum of this generated spike train presents an increased peak at *f*_0_ relative to the baseline. The SNR of these simulated neurons varies linearly as a function of the base firing rate of the neuron (Fig 1H). As a result, the detection of significant oscillations crossing a specific SNR threshold is not possible for a neuron with a low baseline firing rate (Fig 1E), compared to neurons with higher firing rates (Fig 1F–1G), which have a higher SNR and are therefore identified as oscillatory.

**Fig 2:**
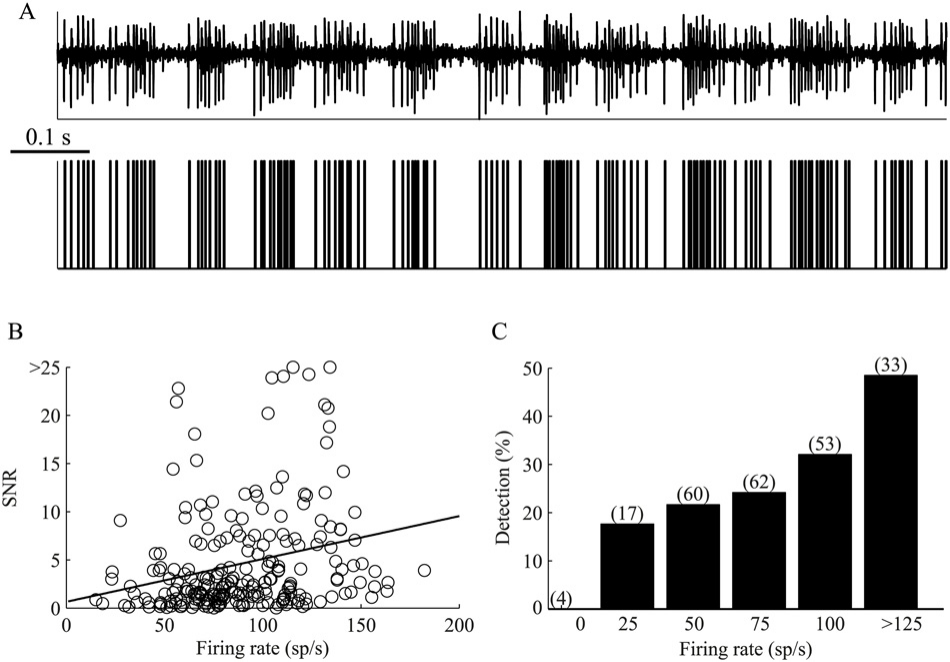
Spike train oscillations—Experimental results. GPi neurons (n = 229) recorded in MPTP treated non-human primates that display typical beta band oscillations (A) Extracellular high pass filtered signal depicting a single GPi neuron (top), and the extracted spike train (bottom). (B) The effect of the firing rate on the SNR (R^2^ = 0.06, p <0.001). (C) Spectral peak detection as a function of the firing rate. The significance level for peak detection was set to 5 STDs. The number of neurons in each group is shown in brackets.

Simulated neurons can illustrate the potential firing rate induced bias in the assessment of spectral activity. To examine the effect of the mean firing rate on the detection of oscillations in experimentally recorded data, we calculated the power spectrum of globus pallidus internus (GPi) neurons recorded in four non-human primates (NHPs) following injections of 1-methyl-4-phenyl-1,2,3,6-tetrahydropyridine (MPTP) which led to the appearance of Parkinsonian symptoms. The firing pattern of a large proportion of the GPi neurons in the Parkinsonian NHP resembles Poissonian firing, which is modulated in an oscillatory manner at a base frequency in the beta band (10–15 Hz) [12–14] (Fig 2A). In the experimental dataset (229 neurons), which consisted of neurons with firing rate in the wide range of 20 to 180 spikes/s and a positive corrected SNR value, the SNR of the highest peak within the beta band varied considerably but exhibited a linear relation with the firing rate (Fig 2B). Thus, the fraction of neurons identified as oscillatory; i.e., neurons with a SNR≥5, increased with the firing rate of the neurons (Fig 2C). This implies that in real experimental data, naïve usage of the power spectrum results in a biased detection of oscillatory activity that can easily lead to misinterpretation of the experimental dataset.

**Fig 3:**
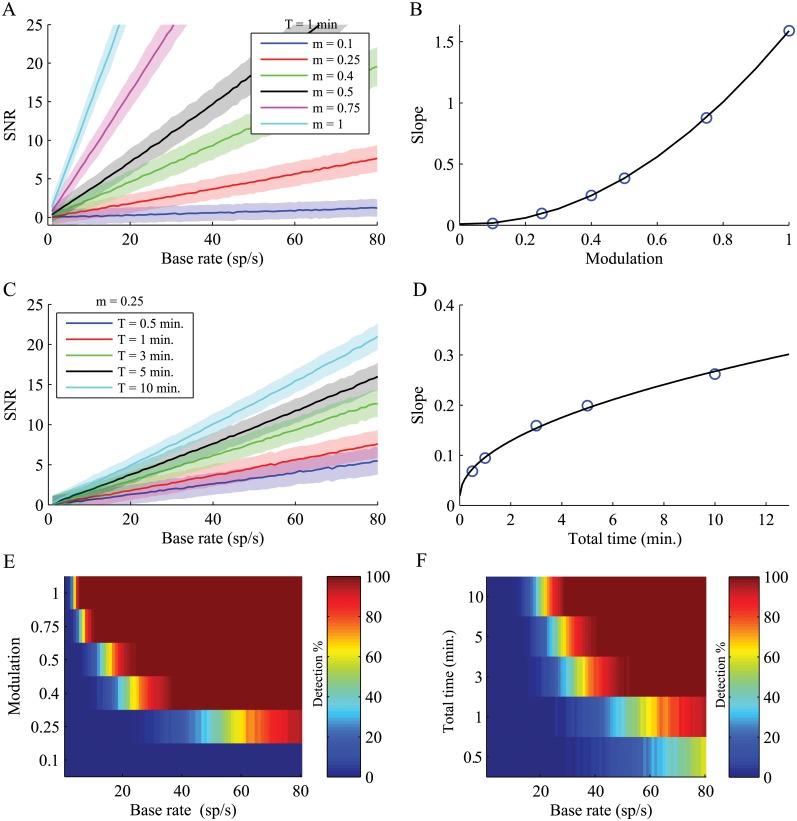
Factors determining the spectral peak magnitude. (A) Effect of the rate modulation (*m*) in different base firing rates (*r*_0_) over a fixed recording period (*T* = 1 minute). The solid lines indicate the mean SNRs; the shaded areas indicate ±1 STD of the SNR. (B) A squared fitting function for the SNR slopes (R^2^ = 1, p <0.001). (C) Effect of the recording period duration *(T)* in different firing rates during a fixed firing modulation (*m* = 0.25). (D) A square root fitting function for the SNR slopes (R^2^ = 0.99). (E-F) The detection of a significant peak (SNR>5) is affected by the rates for different (E) rate modulations and (F) recording durations.

Simulations were next used to quantify the effect of different biological (mean firing rate, oscillation's modulation magnitude) and experimental factors (recording duration) on the SNR. The values of each condition were obtained from 1000 simulated Poisson spike trains generated from a single oscillatory (*f*_0_ = 12 Hz) rate function. When the duration was fixed, and the SNR was estimated over a range of firing rates and modulations, the simulations showed that the SNR had a linear relation to the baseline firing rate (Fig 3A), and a squared relation to the modulation index (Fig 3B). When the modulation index was fixed, and the SNR was estimated over varying firing rates and durations, the simulations indicated a linear relation of the SNR to the firing rate (Fig 3C), and a square root relation to the total length of the recording (Fig 3D). These relations suggest a dependence on these factors that impacts the detection of significant oscillatory activity (Fig 3E–3F). Increases in the firing rate, modulation index (Fig 3E) and recording duration (Fig 3F) result in increased detection of the spectral peak. This dependence on biological and experimental variables thus shows that the ability to objectively detect a peak in the power spectrum is limited. This dependence hinders the comparison of different behavioral states or brain areas and leaves them prone to biases. The formulation of an objective method of measuring oscillations is thus a necessity to enable unbiased comparisons of spike trains arising from different biological and experimental sources.

The power spectrum of an inhomogeneous spike train over a period (*T*) is (see methods: *power spectrum of a finite inhomogeneous Poisson process*):

$$S_{\rho_{T}}\left(f\right)\;=\;\frac{1}{T}\left[\int_{0}^{T}\lambda \left(t\right)dt\;+\;{\left|\int_{0}^{T}\lambda \left(t\right)e^{-i2\pi ft}dt\right|}^{2}\right]$$ (2)

In the specific case of cosine rate modulation over a base frequency (*f*_0_) (Eq 1) the power spectrum is: (see *methods: power spectrum of an inhomogeneous Poisson process with an oscillatory rate function*)

$$\begin{array}{l} S_{\rho_{T}}\left(f\right)\;=\;\\ r_{0}\;+\;r_{0}m\;\text{sinc}(2f_{0}T)\;+\;r_{0}{}^{2}T\;{\text{sinc}}^{2}\;\left(fT\right)\;+\;r_{0}{}^{2}Tm\;\text{sinc}\left(fT\right)\text{sinc}\left[\left(f\;-\;f_{0}\right)T\right]\\ \;+\;r_{0}{}^{2}Tm\;\text{sinc}\left(fT\right)\text{sinc}\left[\left(f\;+\;f_{0}\right)T\right]\;+\;\frac{r_{0}{}^{2}Tm^{2}}{4}\;{\text{sinc}}^{2}\left[\left(f\;-\;f_{0}\right)T\right]\\ +\;\frac{r_{0}{}^{2}Tm^{2}}{4}\;{\text{sinc}}^{2}\left[\left(f\;+\;f_{0}\right)T\right]\;+\;\;\frac{r_{0}{}^{2}Tm^{2}}{2}\text{sinc}\left[\left(f\;-\;f_{0}\right)T\right]\text{sinc}\left[\left(f\;+\;f_{0}\right)T\right]\; \end{array}$$ (3)

The term *sinc(fT)* decays as *1/T*. Thus, for large enough values of *T*, most of the *sinc* terms will decay (e.g., for oscillations at 12 Hz, sinc(2 *f*_0_ *T*) is negligible within a few seconds of recordings), except for the case when the frequency term inside the *sinc* is zero (*i*.*e*. *f-f*_*0*_ = *0*), and then *sinc(0)* equals 1 for every *T*. Consequently, the formulation may be simplified for the case of the base frequency (*f* = *f*_0_); i.e., the peak power, to:

$$S_{\rho_{T}}(f=f_{0})=r_{0}\cdot (1+\;\frac{r_{0}Tm^{2}}{4})\;\;$$ (4)

And for all other frequencies (*f* ≠ *f*_0_); i.e., the baseline power, to:

$$S_{\rho_{T}}(f\neq f_{0})=r_{0}$$ (5)

The magnitude of the peak power and its relation to the baseline power are dependent on multiple factors; namely r_0_, T, m. Thus, a measure that is independent of subjective properties is required which we term the modulation index ($\hat{m}$). This measure can be extracted from the simplified equation of the peak power (Eq 4)

$$\hat{m}=\left|\frac{2\cdot \sqrt{{\hat{S}}_{\rho_{T}}\left(f=f_{0}\right)-{\hat{r}}_{0}}}{{\hat{r}}_{0}\sqrt{T}}\right|$$ (6)

**Fig 4:**
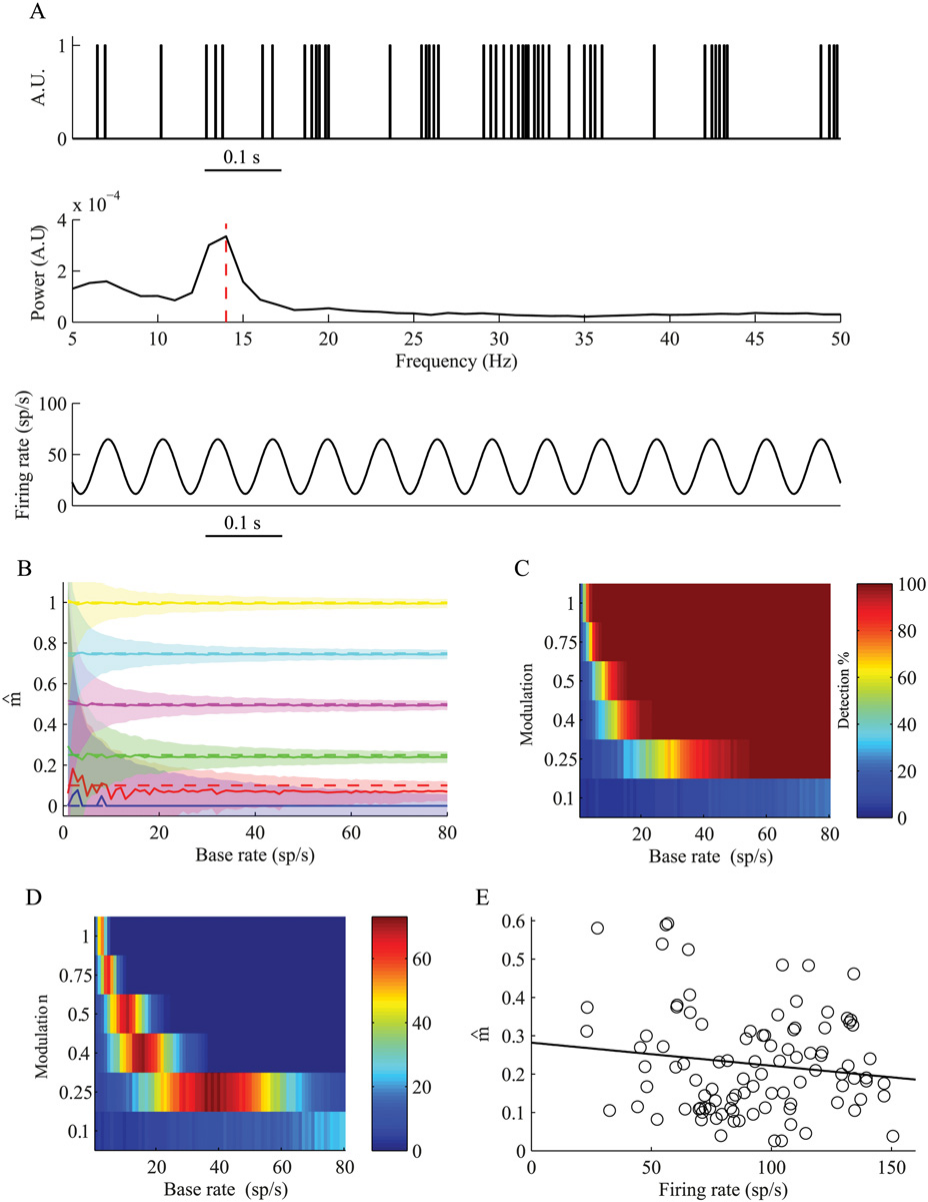
Reconstruction of the rate modulation index. (A) Reconstruction of the rate function of a single GPi neuron. Top—1 second from the neuron's spike train. Middle—power spectrum of the spike train, presenting a peak at ~14 Hz. Bottom—the reconstructed rate function $\hat{\lambda }(t)$. (B) The analytically reconstructed rate modulation index ($\hat{m}$), as a function of the estimated baseline firing rate *(${\hat{r}}_{0}$)*. The solid lines indicate the mean estimated modulation; the shaded areas indicate ±1 STD of the reconstructed modulation, and the dashed line indicates the original modulation. (C) The modulation index detection. The significance level was set according to the simulation results in B; i.e., the mean result for the modulation index of zero at a specific firing rate + 2 STDs. (D) Comparison of the difference between the detections by the modulation index and the spectral peak. (E) Reconstruction of the modulation index of GPi neurons. The results are shown for 98 (of 229) neurons that had a modulation index greater than zero (R^2^ = 0.02, p > 0.1).

This equation enables the extraction of the modulation index for any frequency. When there is a real underlying oscillation in that frequency, the outcome of the equation will be the described modulation index. However, when there is no underlying oscillation in that frequency, the result will tend to be zero, as the value of *S*_*ρ*_*T*__ *(f* ≠ *f*_0_) approaches *r*_0_, as shown in Eq 5. Through this measure, we can reconstruct the rate function of an inhomogeneous Poissonian oscillatory spike train (Fig 4A, top). First, the power spectrum is estimated, and the peak power is extracted (Fig 4A, middle). Then, by using the estimated peak power (${\hat{S}}_{\rho_{T}}\left(f\;=\;f_{0}\right)$), the mean firing rate (${\hat{r}}_{0}$) and the total recording time (*T*), the modulation index is extracted, and the estimated rate function ($\hat{\lambda }(t)$) can be fully described (Fig 4A, bottom).

We calculated the modulation index for the simulation described above. For each of the 1000 generated spike trains we calculated the mean peak power, corrected it for comparison with Welch′s estimator, which is a common method for estimating the power spectrum, (see *methods: correction for the power estimated by Welch's method*), and used it for the calculation of the modulation index. Comparison of the estimated modulation and the original modulation demonstrated that the modulation index was constant across a wide range of parameters over the simulated data (Fig 4B). The significance level for detecting oscillatory neurons using the modulation index depends on the firing rate, and is defined as the mean result for the modulation index of zero at a specific firing rate + 2 standard deviations, as revealed by the simulations. The results of the detection of oscillatory neurons using the modulation index indicate that it is dependent on the baseline rate, such that the detection is better for higher firing rates (Fig 4C). For example, when the modulation is 0.4, and the required detection probability is 80%, the firing rate should be higher than 15 spikes/s. However, for all firing rates, the detection using the modulation index was better than the detection using the spectral peak (Fig 4D). We applied the modulation index to the recordings from the GPi of the Parkinsonian NHP (Fig 4E) and obtained a constant modulation index ($\hat{m}\;=\;0.23\pm 0.13\;$ mean ± SD). In our experimental dataset, 98 neurons were modulated; i.e., had a modulation index greater than zero, and 78 of these were significant, according to the aforementioned significance test. The results for all neurons revealed that the oscillation modulation was independent of the firing rate (R^2^ = 0.02, p >0.1), unlike the results obtained using the power spectrum (Fig 2B).

**Fig 5:**
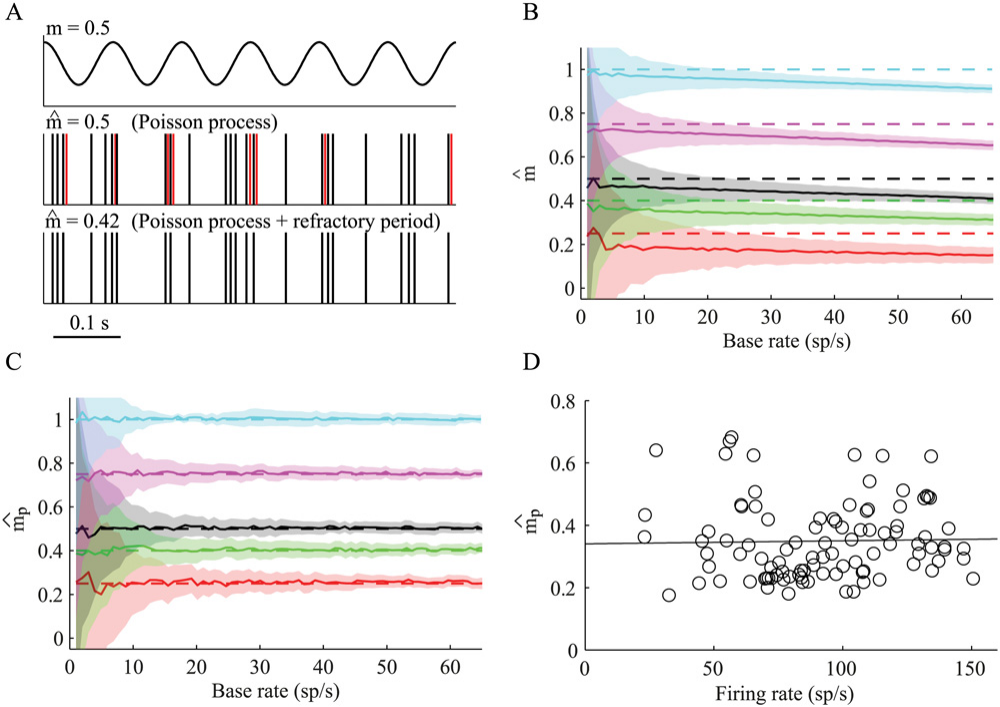
Correction of the modulation index for deviations from the Poisson model. (A) A short segment (0.5 s) taken from: Top—an oscillatory rate function (*f*_0_ = 12 Hz, *r*_0_ = 60 sp/s and *m* = 0.5). Middle—an example of a Poisson generated spike train from this rate function, without refractory period. Bottom—an example of a Poisson generated spike train with refractory period of 2 ms. The red lines in the middle spike train indicate the missing spikes in the bottom spike train, due to the refractory period. (B) The analytically reconstructed modulation index ($\hat{m}$), as a function of the estimated baseline firing rate (${\hat{r}}_{0}$), for spike trains with refractory period. The solid lines indicate the mean estimated modulation; the shaded areas indicate ±1 STD of the reconstructed modulation, and the dashed line indicates the original modulation. (C) The corrected modulation index ($\hat{m_{p}}$) for the same spike trains in B. (D) Reconstruction of the modulation index of GPi neurons after correction of the modulation index. The black line is the linear regression for all the neurons (n = 98, R^2^ = 0.0005, p >0.5).

The modulation index was derived above for an inhomogeneous Poisson spike train where the power spectrum exhibits equal power in all frequencies except for the base frequency. However, real neurons deviate from the Poisson model, primarily as a result of the refractory period. Refractoriness prevents the neuron from firing two successive spikes within a short interval, and thus the spike train is never a true Poisson process. In the case of an oscillatory spike train, refractoriness distorts the oscillation, and the modulation appears to be smaller than the real modulation of its rate function (Fig 5A). This occurs due to the larger effect of the refractory period around the peak of the oscillation as more spikes are expected at that time. This underestimation of the modulation index is more prominent for high firing rates due to the increased effect of the refractory period (Fig 5B). The modulation index can however be corrected to accommodate for the refractory period (see methods). The correction procedure reconstructs the modulation index ($\hat{m_{p}}$) of the rate function that will result in the analytically calculated modulation index, which is smaller than expected as a result of the refractory period, and thus extracting the "true" modulation of the driving rate function (Fig 5C). We applied this correction to GPi spike trains. This resulted in higher modulation index values ($\hat{m_{p}}\;=\;0.35\pm 0.12\;$ mean ± SD) that are independent from the firing rate (R^2^ = 0.0005, p >0.5) (Fig 5D). The percentage of significantly modulated neurons (97/229, 42%) was significantly larger than the percentage of neurons that were identified as significantly oscillatory by the SNR measure (64/229, 28%) (*x* ^2^ test, *p* < 0.01).

The detection of oscillatory activity depends on a critical experimental factor—the total recording duration *(T)*. This factor must be set prior to the experiment itself to avoid a case of failed detection due to the experiment′s time constraint (Fig 3F) rather than an actual lack of oscillations. The analogous analytic SNR is defined as:

$$SNR\left(f\right)\;=\;\frac{\int_{0}^{T}\lambda \left(t\right)e^{-i2\pi ft}dt}{\int_{0}^{T}\lambda \left(t\right)\;dt}$$ (7)

And for *f* = *f*_0_:

$$\begin{array}{l} SNR\left(f=f_{0}\right)\;=\;\\ {}[r_{0}{}^{2}T^{2}{\text{sinc}}^{2}\;\left(f_{0}T\right)\;+\\ r_{0}{}^{2}T^{2}m\text{ sinc}\left(f_{0}T\right)\;+\;r_{0}{}^{2}T^{2}m\text{ sinc}\left(f_{0}T\right)\text{ sinc}\left(2f_{0}T\right)\;+\;\frac{r_{0}{}^{2}T^{2}m^{2}}{4}\;+\\ \frac{r_{0}{}^{2}T^{2}m^{2}}{4}\;{\text{sinc}}^{2}\left(2f_{0}T\right)\;+\;\frac{r_{0}{}^{2}T^{2}m^{2}}{2}\text{sinc}\left(2f_{0}T\right)\;]/\\ \left[r_{0}T\;+\;r_{0}Tm\text{ sinc}(2f_{0}T)\right] \end{array}$$ (8)

And for large enough T:

$$SNR(f=f_{0})=\;\frac{r_{0}Tm^{2}}{4}$$ (9)

The expected analytic SNR and the numerical estimated $\hat{SNR}$ are similar, so we can replace the analytic *SNR* with the desired $\;\hat{SNR}$, such that:

$$\hat{SNR}=\;\frac{{\hat{r}}_{0}T{\hat{m}}^{2}}{4}$$ (10)

This equation is true when no windows are used. When using Welch's method, the SNR must be multiplied by the root of the number of windows used (see *methods: effect of the window size on the SNR*), which is defined as:

$$N_{wins}=T/wl$$ (11)

where *wl* is the window length, and *N*_*wins*_ is the number of windows used.

From this equation, the required time for significance of the $\hat{SNR}$ is:

$$\hat{T}\;=\;\frac{16\cdot {\hat{SNR}}^{2}}{wl\cdot {\hat{r}}_{0}{}^{2}{\hat{m}}^{4}}$$ (12)

**Fig 6:**
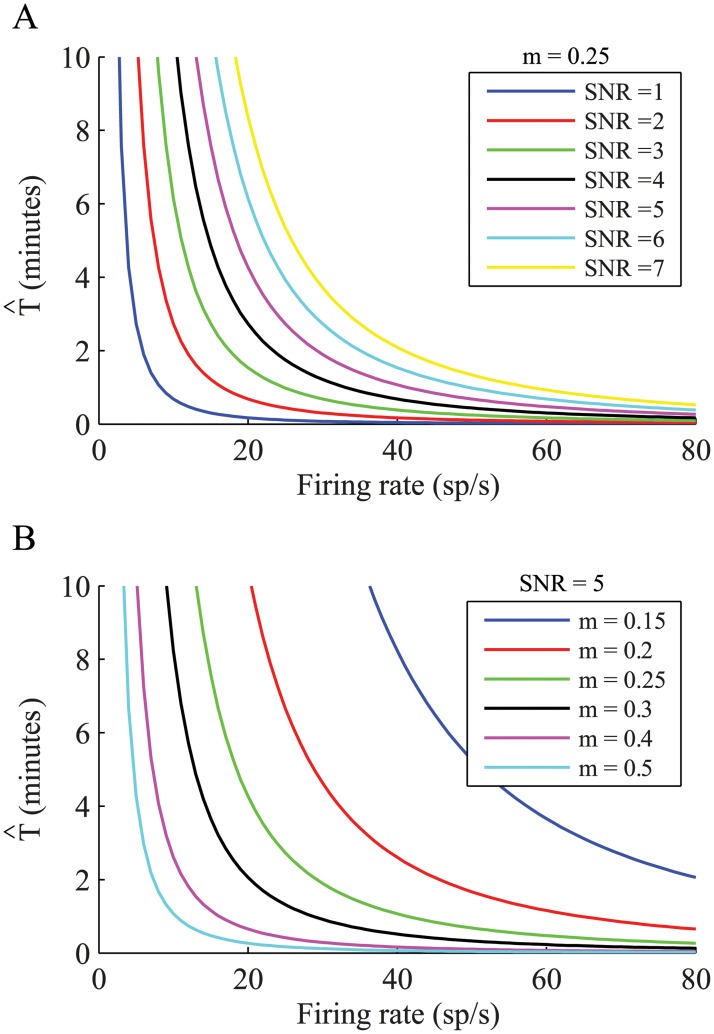
Estimating the required recording duration. Analytic results for the required recording duration, (A) over different firing rates and for increasing SNR, with modulation of 0.25 and (B) over different firing rates and for various modulations, calculated for a SNR of 5.

We calculated the required recording time for a window length of 1 second for various firing rates, for a $\hat{SNR}$ of 1 to 7 while the modulation was fixed at 0.25 (Fig 6A), and for a fixed $\hat{SNR}$ of 5 for various modulations (Fig 6B). High firing rates as well as high modulation index values will result in a shortening of the required recording duration. In addition, setting the significance of the SNR to lower values will shorten the required recording duration. Thus, for example, for a GPi neuron with a mean firing rate of 75 spike/s and an estimated modulation index of 0.25, less than half a minute of recording will suffice to detect a spectral peak with SNR of 5. However, in order to detect a spectral peak with SNR of 7 for the same neuron, about 1 minute of recording is necessary.

## Discussion

Classical oscillation estimation methods for neuronal data represent the magnitude of oscillatory activity in terms of elevated peaks in a specific frequency, as compared to the baseline defined over other frequencies. In the first part of this study we quantified the dependence of the magnitude of the spike train oscillation, as revealed by power spectrum estimation, on different biological and experimental factors. We showed that in both simulated and experimental data, the estimated magnitude of the oscillation depends highly on the mean firing rate of the spike train. We used simulations to quantify this dependence and investigated the influence of these factors on the detectability of oscillatory spike trains.

In the second part of this study, we introduced a novel method for assessing the oscillatory nature of a spike train by calculating the modulation index of the oscillation. The modulation index can be estimated on the basis of the mean firing rate, the total recording duration, and the magnitude of the peak in the power spectrum, and provides an objective measure of the oscillation magnitude. We applied this measure to the simulated spike trains, and showed that the measure produces a correct estimation over a wide range of biologically plausible parameters. The application of this measure to experimental data recorded from GPi neurons in the Parkinsonian NHP revealed that the modulation index is independent of the firing rate. We introduced the corrections that need to be applied to the estimated power revealed by Welch's method, to compare the index to the analytically calculated power. Furthermore, we presented adaptations to the modulation index to account for deviations from the Poisson process assumptions. Explicitly, we show its usage in an inhomogeneous Poisson process with an absolute refractory period, which dramatically alters the spectrum of real neurons. Finally, we proposed a practical method for estimating the recording time required to accurately detect oscillations in neurophysiological experiments.

The effect of the firing rate on the size of the spectral peak is dramatic. In the mammalian brain, the range of firing rates between brain areas and neuronal types is considerable, and even within a specific brain region and a specific neuronal type, the heterogeneity of firing rates within the population is large. For example, the firing rates of the GPi neurons in the Parkinsonian NHP used in our dataset ranged from 25 spikes/s to over 100 spikes/s. Furthermore, the rate distribution can vary between behavioral states: in the normal state, firing rates increase when attention is directed toward a stimulus [15] and changes in the firing rates of hippocampal neurons occur as the result of experience [16]. Pathological conditions influence firing rates as well, as shown in the firing rate changes throughout the basal ganglia over the course of Parkinson's disease [17,18]. These differences consequently lead to a situation in which identification of oscillations using the spectral peak may be difficult in some conditions, and moreover, bias the comparison across different brain areas, neuron types and behavioral states. Nonetheless, most studies do not take different firing rates into account when inferring their spectral results. For example, previous studies of the Parkinsonian primate have shown that there is a larger fraction of oscillating neurons as well as an increased magnitude of oscillations in the GPi relative to the globus pallidus externus (GPe) [14,19]. These studies neglected to take the substantially different firing rates between the two brain areas during parkinsonism into account: the mean firing rate of GPe neurons in the MPTP treated NHP is 45 spikes/s, whereas the mean firing rate of the GPi neurons is 75 spikes/s [19]. According to our simulation results (Fig 3E), the detection of significant oscillations in neurons with a realistic modulation index of 0.25 and a firing rate of 45 spikes/s is less than 20%, whereas when the firing rate is 75 spikes/s the detection is about 80%. Thus, the conclusions relating to different oscillatory activities may be derived from the firing rates themselves and not from the oscillatory nature of the neurons.

The firing rate effect on the spectral peak is also a major caveat in the interpretation of the oscillatory nature of MUA. Previous studies have reported that MUA appears to be more oscillatory than single-unit activity (SUA) [14,20]. However, this phenomenon is affected to a large extent by the higher firing rate of the MUA that contains more than one spike train, such that the comparison between the spectral peak of SUA and MUA is problematic and may lead to a misinterpretation of the results.

An additional factor that biases the spectral peak estimation considerably is not dependent on the neuronal properties but rather on an experimental property: the duration of the recording. As the total recording duration increases, the SNR will increase. Yet, most studies are not aware of this bias when comparing the results of spectral analyses of recordings with different durations. Moreover, in some situations, the duration of the recording can be extremely short, such as recordings in the operation room which can only yield a few seconds of recording [20], and thus are not long enough for the detection of a spectral peak despite an underlying oscillatory rate function. Even worse, transient changes in the oscillatory activity of a neuron in response to a behavioral task might prevent the detection of a spectral peak which is unique to the transient period. Such transient changes occur for instance in the gamma frequency in relation to movement [1].

Thus, a measure quantifying oscillations is needed that goes beyond the identification of significant spectral peaks. Several methods for the estimation of oscillatory activity in spike trains have been suggested based on the auto-correlation function [10,21] and analysis of the power spectrum [7]. As the spectrum may be defined as the Fourier transform of the auto-correlation function (Wiener-Khinchin theorem), these two groups of methods are biased. The bias could be visualized by the auto-correlation function of the spike trains as well as in its power spectrum (S1A–S1F Fig). As in the power spectrum, the SNR of the auto-correlation function is dependent on the firing rate, such that when the firing rate is higher, the oscillatory nature of the spike train is more evident in the auto-correlation function, while during low firing rate, the oscillation cannot readily be seen (S1G Fig). In order to overcome the bias in the auto-correlation, one of the methods defines an oscillatory score that is less sensitive to the firing rate [10] but still depends on the frequency band, where higher bands yield higher scores. Other methods have dealt with the problem of finite recording durations by applying corrections to the confidence limits of the spectral estimations [7] but have not handled the different firing rates explicitly.

The major drawback in the standard methods of assessing spike train oscillations were addressed in consecutive steps within this manuscript. Initially, we quantified the factors that bias the magnitude of the spectral peak. Next, we introduced a measure that overcomes these factors, and leads to a reliable detection of oscillations and a direct estimation of the strength of oscillations: the modulation index. This method is simple and fast, and can be customized to accommodate the size of the window and the spectral smoothing applied prior to the spectral estimation. Finally, a practical method for estimating the required recording duration was proposed, based on the mean firing rate, the evaluated modulation index and the desirable SNR. In order to evaluate the mean firing rate and the modulation index, a few short preliminary recordings can be performed, and these two parameters can be grossly assessed. The significance levels for the SNR have to be chosen, and then, the required recording duration may be estimated. In other situations, where the recording duration is fixed, and cannot be adjusted, the experimenter should be aware of the limitations of detecting oscillations from the recordings and use our analytic results to estimate the fraction of unidentified oscillatory neurons.

Throughout most of this study we assumed that the spiking activity of single neurons follows a Poisson distribution [22–24], i.e. they fire stochastically and the probability of generating a spike at a certain time depends solely on the underlying rate function. In real neurons, there are deviations from the Poisson model. The most prominent deviation, the refractory period, was addressed in this study by correcting the estimation of the modulation index. The correction procedure is based on information extracted from the given spike train, and on the underlying rate function, including the deviation from the Poisson process. The process numerically finds the original modulation index of the rate function that results in the modulation index calculated analytically from the recorded data. For the case of refractory period, we modeled the rate function with an absolute refractory period following a spike. However, in some unique cases, when the refractory period causes the oscillation frequency peak to be too small, which results in a modulation index of zero, the correction procedure will not be able to reveal the original modulation. In this situation, a shuffling procedure on the first order ISI's could compensate for the distortion of the spectrum caused by the refractoriness and lead to an increased accuracy of the peak detection [25]. The general rate function model could be expanded to accommodate for other deviations from the Poisson model, such as relative refractory period and bursting activity; i.e., an elevated firing probability immediately after the refractory period.

In conclusion, the modulation index can provide an objective quantification of spike train oscillations, and thus an unbiased comparison across brain regions, behavioral states and separate recordings with different recording lengths. Using this quantification method can expand our understanding of neural oscillations, and their role in normal and pathological states.

All the code required for the estimation of the modulation index and the required recording time is available as custom MATLAB code (compatible with versions 2007B-2014A) on our website: http://neurint.ls.biu.ac.il/software/

## Methods

### Ethics statement

The neuronal recordings were taken from four Cynomologus monkeys (Macaca Fascicularis), that underwent 1-methyl-4-phenyl-1,2,3,6-tetrahydropyridine (MPTP) injections leading to a stable Parkinsonian state. The monkeys’ water and food consumption and weight were checked daily and their health was monitored by a veterinarian. All procedures were in accordance with the National Institutes of Health Guide for the Care and Use of Laboratory Animals, Bar-Ilan University Guidelines for the Use and Care of Laboratory Animals in Research and the recommendations of the Weatherall Report. All procedures were approved and supervised by the Institutional Animal Care and Use Committee (IACUC). These procedures were approved by the National Committee for Experiments on Laboratory Animals at the Ministry of Health (permit number BIU150605). Full details of the experimental protocol appear elsewhere [14,26].

### Spike train simulations

The spike trains were simulated with a resolution of Δt = 1 ms. The spike trains were modeled as an inhomogeneous Poisson process, whose rate is modulated by a 12 Hz cosine function. Spike trains with refractory period were modeled by setting the instantaneous firing rate to zero for 2 ms following a spike.

### Power spectrum

The power spectrum was estimated based on Welch's method [27] for spectral estimation using non-overlapping segments. In all spectral calculations, the 1000 bin segments (see *methods: Effect of the window size on the SNR*) were windowed using a Hamming window. The power spectral densities of each segment were calculated, and were then averaged. The bin size (Δt) of both the experimental and simulated data was 1 ms, leading to a spectral resolution of 1 Hz and a maximal frequency of 500 Hz.

### Power spectrum of a finite inhomogeneous Poisson process

The following is based on the general formulation by Pinhasi and Lurie [28]: Let's consider a Poisson process of occurring delta functions, with a non-constant and time dependent rate λ(*t*). A portion of the process realization in the time interval [0 *T*] is given by the finite summation:

$$\rho_{T}\left(t\right)=\sum_{i=1}^{k(t)}\delta (t-t_{i})$$ (13)

where *t*_*i*_ are the successive firing instances, *k*(*t*) is a random variable that counts the number of delta functions occurring during the time interval T, with an expected value of:

$$\bar{k\left(T\right)}=\;\int_{0}^{T}\lambda \left(t\right)dt$$ (14)

The event appearance *t*_*i*_ is an independent random variable with the probability density function:

$$f\left(t_{i}\right)\;=\;\frac{\lambda \left(t_{i}\right)}{\bar{k\left(T\right)}}=\;\;\frac{\lambda \left(t_{i}\right)}{\int_{0}^{T}\lambda \left(t\right)dt}$$ (15)

The Fourier transform of *ρ_T_*(*t*) is:

$$\rho_{T}\left(f\right)\;=\;\sum_{c=1}^{k(T)}e^{i2\pi ft_{c}}$$ (16)

The power spectral density can be calculated:

$$\begin{array}{l} S_{\rho_{T}}\left(f\right)\;=\;\;\frac{{\left|\bar{\rho_{T}\left(f\right)}\right|}^{2}}{T}\;=\;\;\frac{1}{T}\bar{\sum_{c=1}^{k(T)}\sum_{c^{\prime }=1}^{k(T)}e^{i2\pi f\left(t_{c}-\;t_{c^{\prime }}\right)}}\\ \;=\;\frac{1}{T}\left[\bar{\sum_{c=1}^{k\left(T\right)}1}\;+\;\bar{\sum_{c=1}^{k\left(T\right)}\sum_{c^{\prime }\neq c}^{k\left(T\right)}e^{i2\pi f\left(t_{c}-\;t_{c^{\prime }}\right)}}\;\right]\\ \;=\;\frac{1}{T}\;\left\{\bar{k\left(T\right)}\;+\;\bar{k\left(T\right)\left[k\left(T\right)-1\right]}\;\cdot \;\bar{e^{i2\pi f\left(t_{c}-\;t_{c^{\prime }}\right)}}\right\}\\ \;=\;\frac{1}{T}\;\left\{\bar{k\left(T\right)}\;+\;\left[\bar{k^{2}\left(T\right)}-\;\bar{k\left(T\right)}\right]\;\cdot \;\bar{e^{i2\pi f\left(t_{c}-\;t_{c^{\prime }}\right)}}\right\} \end{array}$$ (17)

In a Poisson process: ${\sigma_{k}}^{2}\left(T\right)\;=\;\overset{-}{k\left(T\right)}$, and thus, the second moment is:

$$\bar{k^{2}\left(T\right)}\;=\;{\left[\bar{k\left(T\right)}\right]}^{2}\;+\;\sigma_{K}{}^{2}\left(T\right)\;=\;{\left[\bar{k\left(T\right)}\right]}^{2}\;+\;\bar{k\left(T\right)}.$$ (18)

Inserting this in Eq 17 yields:

$$S_{\rho_{T}}\left(f\right)\;=\;\frac{1}{T}\;\left\{\bar{k\left(T\right)}\;+\;{\left[\bar{k\left(T\right)}\right]}^{2}\;\cdot \;\bar{e^{i2\pi f\left(t_{c}-\;t_{c^{\prime }}\right)}}\right\}$$ (19)

The statistical average:

$$\bar{e^{+i2\pi ft_{c}}}\;=\;{\left(\bar{e^{-i2\pi ft_{c^{\prime }}}}\right)}^{*}\;=\;\int_{0}^{T}e^{+i2\pi ft_{c}}\;f\left(t_{c}\right)dt_{c}\;=\;\frac{1}{\bar{k\left(T\right)}}\int_{0}^{T}\lambda \left(t_{c}\right)e^{+i2\pi ft_{c}}\;dt_{c}$$ (20)

Leading to the power spectrum formulation as:

$$S_{\rho_{T}}\left(f\right)=\;\frac{1}{T}\left[\int_{0}^{T}\lambda \left(t\right)dt+\;{\left|\int_{0}^{T}\lambda \left(t\right)e^{-i2\pi ft}dt\right|}^{2}\right]$$ (21)

where the first term in brackets is the statistical average of the number of events over T, and the second term is the power spectrum of the instantaneous rate function λ(*t*) in the interval [0 *T*].

### Power spectrum of an inhomogeneous Poisson process with an oscillatory rate function

For a rate function defined as:

$$\lambda (t)=r_{0}\cdot (1+m\cdot \text{cos}(2\pi \cdot f_{0}\cdot t))$$ (22)

The expected number of spikes within a period *T* is:

$$\bar{k\left(T\right)}\;=\;\int_{0}^{T}\lambda \left(t\right)dt\;=\;r_{0}T\left[1\;+\;m\text{ sinc}(2f_{0}T)\right]$$ (23)

The Fourier transform of the instantaneous rate in the time interval [0 *T*] for a large *T* is:

∫0Tλ(t)e−i2πftdt= ∫−T2T2[r0+r0mcos(2πf0t)]e−i2πftdt=∫−T2T2r0e−i2πftdt+ ∫−T2T2r0m2e−i2π(f−f0)tdt  + ∫−T2T2r0m2e−i2π(f+f0)tdt=r0Tsinc(fT)+ r0mT2sinc[(f−f0)T]+ r0mT2sinc[(f+f0)T] (24)

And:

$$\begin{array}{l} {\left|\int_{0}^{T}\lambda \left(t\right)e^{-i2\pi ft}dt\right|}^{2}\;=\;r_{0}{}^{2}T^{2}\;{\text{sinc}}^{2}\;\left(fT\right)\;+\;r_{0}{}^{2}T^{2}\;m\;\text{sinc}\left(fT\right)\;\text{sinc}\left[\left(f\;-\;f_{0}\right)T\right]\\ +\;r_{0}{}^{2}T^{2}\;m\;\text{sinc}\left(fT\right)\text{sinc}\left[\left(f\;+\;f_{0}\right)T\right]\;+\;\;\frac{r_{0}{}^{2}T^{2}m^{2}}{4}\;{\text{sinc}}^{2}\left[\left(f\;-\;f_{0}\right)T\right]\;\\ \;+\;\;\frac{r_{0}{}^{2}T^{2}m^{2}}{4}\;{\text{sinc}}^{2}\left[\left(f+f_{0}\right)T\right]\;+\;\frac{r_{0}{}^{2}T^{2}m^{2}}{2}\text{sinc}\left[\left(f\;-\;f_{0}\right)T\right]\text{sinc}\left[\left(f\;+\;f_{0}\right)T\right]\; \end{array}$$ (25)

Resulting in the power spectrum of the inhomogeneous Poisson process:

$$\begin{array}{l} S_{\rho_{T}}\left(f\right)\;=\;\frac{1}{T}\left[\int_{0}^{T}\lambda \left(t\right)dt\;+\;{\left|\int_{0}^{T}\lambda \left(t\right)e^{-i2\pi ft}dt\right|}^{2}\right]\;=\\ \;r_{0}\;+\;r_{0}m\;\text{sinc}(2f_{0}T)\;+\;r_{0}{}^{2}T\;{\text{sinc}}^{2}\;\left(fT\right)\;+\;\;r_{0}{}^{2}Tm\;\text{sinc}\left(fT\right)\text{sinc}\left[\left(f\;-\;f_{0}\right)T\right]\\ \;+\;r_{0}{}^{2}Tm\;\text{sinc}\left(fT\right)\text{sinc}\left[\left(f\;+\;f_{0}\right)T\right]\;\;+\;\frac{r_{0}{}^{2}Tm^{2}}{4}\;{\text{sinc}}^{2}\left[\left(f\;-\;f_{0}\right)T\right]\\ +\;\frac{r_{0}{}^{2}Tm^{2}}{4}\;{\text{sinc}}^{2}\left[\left(f\;+\;f_{0}\right)T\right]\;+\;\frac{r_{0}{}^{2}Tm^{2}}{2}\text{sinc}\left[\left(f\;-\;f_{0}\right)T\right]\text{sinc}\left[\left(f\;+\;f_{0}\right)T\right]\; \end{array}$$ (26)

### Correction for the power estimated by Welch's method

The power as estimated by Welch′s method, which calculates the average of the spectra of windowed segments, needs to be scaled to the analytically calculated power; i.e., the power calculated for the signal as a single non-windowed segment. To do so, Welch's power needs to be multiplied first by the sampling frequency, and then divided by 2. Then, a correction due to the lower spectral resolution of Welch's power needs to be made: first multiply the power by the number of windows, and then subtract from it the baseline power multiplied by the number of windows minus 1. Finally, a correction due to the windowing needs to be applied: the correction term is the mean of the window multiplied by the length of the window, and then divided by the power of the window. This is expressed in the final formulation:

$$Peak_{analytic}=[(Peak_{Welch}\cdot \frac{Fs}{2})\cdot N_{wins}-(N_{wins}-1)\cdot r_{0}]\cdot [mean(w)\cdot wl/(w^{'}\cdot w)]$$ (27)

where *Fs* is the sampling frequency, *N*_*wins*_ is the number of windows used, *w* is the window, and *wl* is the length of the window.

### Correction of the modulation index for deviations from the Poisson model

The deviation of the spike train from the Poisson model results in an incorrect estimation of the modulation index. A correction process could compensate for this: (1) The firing rate of the spike train assuming a true Poisson process ($\hat{r_{p}}$) is calculated using the recorded spike train. (2) An oscillatory rate function is generated of length *T* and baseline rate which is set to $\hat{r_{p}}$. The modulation of the rate function (*m*_*p*_) is set to the analytically calculated modulation index ($\hat{m})$ of the spike train. (3) From this rate function, multiple new Poisson-like spike trains are generated, including the given deviations from the Poisson model. The modulation index measure of each spike train is estimated (${\hat{m}}_{s}$), and the mean modulation index is calculated ($\overset{-}{{\hat{m}}_{s}}$). (4) The mean simulated modulation index is compared to the original modulation index of the spike train. If the simulated modulation index is different from the original modulation index ($\overset{-}{{\hat{m}}_{s}}\neq \hat{m}$), increase (in the case of an underestimation) or decrease (in the case of an overestimation) the modulation of the rate function (*m*_*p*_), and repeat step 3. If the simulated modulation index is similar to the original modulation index ($\overset{-}{{\hat{m}}_{s}}\;=\;\hat{m}$) within the required error boundaries, the Poisson equivalent modulation index is the modulation of the rate function (${\hat{m}}_{p}$).

### Correction of the modulation index for the refractory period

The correction process was explicitly implemented to compensate for the underestimation of the modulation index arising from the refractory period: (1) The firing rate of the spike train assuming no refractory period is calculated using the properties of the recorded spike train: the number of spikes (*N*_*spikes*_), the recording duration (*T*) and the refractory period (*τ*_*ref*_), which could be extracted from the interspike interval (ISI) histogram:

$$\hat{r_{p}}=N_{spikes}/(T-\tau_{ref}\cdot N_{spikes})$$ (28)

(2) The oscillatory rate function is generated of length *T* using a baseline rate ($\hat{r_{p}}$) and the modulation index ($\hat{m})$ calculated from the spike train. (3) From this rate function, 100 new Poisson spike trains are generated using a refractory period of *τ*_*ref*_, and their mean modulation index is calculated ($\overset{-}{{\hat{m}}_{s}}$). (4) If the simulated modulation index is smaller than the original modulation index ($\overset{-}{{\hat{m}}_{s}}<\hat{m}$), increase the modulation of the rate function (*m*_*p*_), and repeat step 3. If the simulated modulation index is similar to the original modulation index ($\overset{-}{{\hat{m}}_{s}}\geq \hat{m}$), the corrected modulation index is the modulation of the rate function ($\hat{m_{p}}$).

### Effect of the window size on the SNR

**Fig 7:**
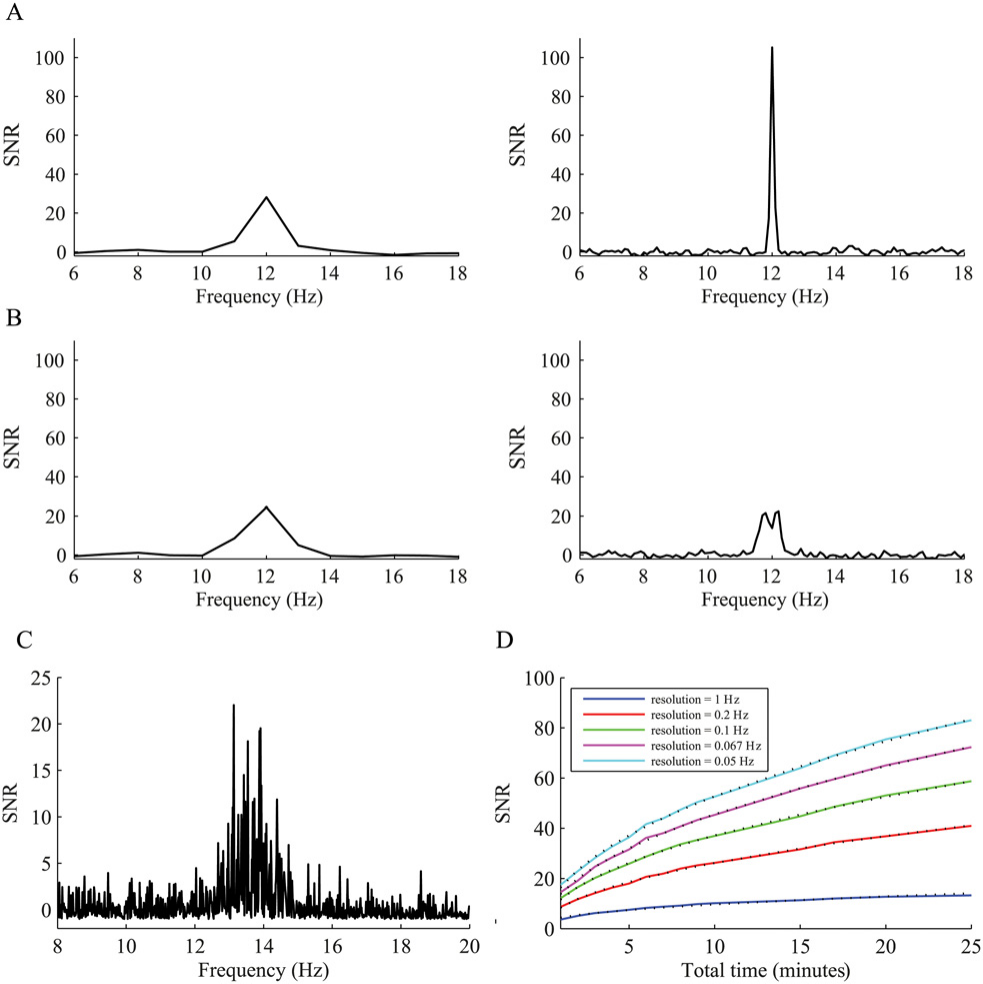
Selection of window size for the calculation of the power spectrum. (A) Power spectrum of a Poisson spike train generated from a pure oscillatory rate function (*f*_0_ = 12 Hz, *r*_0_ = 40 sp/s and *m* = 0.5). Left, power spectrum calculated with a resolution of 1 Hz. Right, power spectrum with a resolution of 0.1 Hz. (B) Power spectrum of a Poisson spike train generated from a jittered oscillatory rate function (*f*_0_ = 11.5–12.5 Hz, *r*_0_ = 40 sp/s and *m* = 0.5). Left, 1 Hz resolution. Right, 0.1 Hz resolution. (C) An example of a single GPi neuron showing a peak with a width of ~1 Hz. (D) The effect of window size and resolution in the power spectrum at different recording durations during a fixed firing rate and a fixed firing modulation. The SNR increases on a scale of $\sqrt{N_{wins}}$. The dotted black lines indicate the square root fit.

The window size must be chosen as a function of the resolution of the frequency of the underlying oscillation. When the frequency of the oscillation is stable and tightly locked to a specific frequency, a longer window will yield a more precise power spectrum (Fig 7A). However, when the frequency jitters, using a window with a resolution smaller than the jitter size will not lead to a more precise spectrum, but rather to a spread of the power over the entire frequency jitter range (Fig 7B). Real neurons are not perfect oscillators in a single precise frequency, but instead are oscillatory within a jittered frequency range, which in the GPi of the Parkinsonian NHP is about 1 Hz (Fig 7C), justifying a window size of around the jitter size (a window length of 1000 leads to a 1Hz resolution). The length of the window affects the SNR, such that the SNR grows like $\sqrt{N_{wins}}\;=\;\sqrt{\frac{T}{wl}}\;$(Fig 7D).

## Supporting Information

S1 Fig Reflection of the oscillatory activity in the auto-correlation function and the ISI histogram. Normalized power spectrum of 5 minutes simulated spike train generated from an oscillatory rate function (*f*_0_ = 12 Hz, *m* = 0.5), when the base rate is (A) 5 sp/s, (B) 15 sp/s, (C) 30 sp/s. (D-F) The auto-correlation function of the same spike trains as in A-C. (G) The SNR of the peak in the auto-correlation as a function of the mean firing rates. The SNR is defined as the number of STDs from the mean of the auto-correlation function to the first peak near the zero point. The circles indicate the SNR of the peak shown in examples D-F, and the solid line indicates the fitted linear function. (H-J) The first order ISI histograms of the same spike trains shown in A-C. The dashed vertical lines indicate the 1/*f*_0_ ISI.
(DOCX)

## References

[1] Engel a K, Fries P, Singer W (2001) Dynamic predictions: oscillations and synchrony in top-down processing. Nat Rev Neurosci 2: 704–716. 11584308 10.1038/35094565

[2] Hutchison WD, Dostrovsky JO, Walters JR, Courtemanche R, Boraud T, et al (2004) Neuronal oscillations in the basal ganglia and movement disorders: evidence from whole animal and human recordings. J Neurosci 24: 9240–9243. 15496658 10.1523/JNEUROSCI.3366-04.2004 PMC6730107

[3] Buzsáki G, Draguhn A (2004) Neuronal oscillations in cortical networks. Science 304: 1926–1929. 15218136 10.1126/science.1099745

[4] Bergman H, Wichmann T, Karmon B, DeLong MR (1994) The primate subthalamic nucleus. II. Neuronal activity in the MPTP model of parkinsonism. J Neurophysiol 72: 507–520. 7983515 10.1152/jn.1994.72.2.507

[5] Gray CM, Singer W (1989) Stimulus-specific neuronal oscillations in orientation columns of cat visual cortex. Proc Natl Acad Sci U S A 86: 1698–1702. 2922407 10.1073/pnas.86.5.1698 PMC286768

[6] Buzsáki G (2006) Rhythms of the Brain. Oxford: Oxford University Press.

[7] Jarvis MR, Mitra PP (2001) Sampling properties of the spectrum and coherency of sequences of action potentials. Neural Comput 13: 717–749. 11255566 10.1162/089976601300014312

[8] Moran a, Bar-Gad I (2010) Revealing neuronal functional organization through the relation between multi-scale oscillatory extracellular signals. J Neurosci Methods 186: 116–129. 10.1016/j.jneumeth.2009.10.024 19900473

[9] Perkel DH, Gerstein GL, Moore GP (1967) Neuronal spike trains and stochastic point processes. I. The single spike train. Biophys J 7: 391–418. 4292791 10.1016/S0006-3495(67)86596-2 PMC1368068

[10] Mureşan RC, Jurjuţ OF, Moca V V, Singer W, Nikolić D (2008) The oscillation score: an efficient method for estimating oscillation strength in neuronal activity. J Neurophysiol 99: 1333–1353. 18160427 10.1152/jn.00772.2007

[11] Nikolic D (2009) Model this! Seven empirical phenomena missing in the models of cortical oscillatory dynamics. Proceedings of the International Joint Conference on Neural Networks. pp. 2272–2279.

[12] Nini A, Feingold A, Slovin H, Bergman H (1995) Neurons in the globus pallidus do not show correlated activity in the normal monkey, but phase-locked oscillations appear in the MPTP model of parkinsonism. J Neurophysiol 74: 1800–1805. 8989416 10.1152/jn.1995.74.4.1800

[13] Raz A, Vaadia E, Bergman H (2000) Firing patterns and correlations of spontaneous discharge of pallidal neurons in the normal and the tremulous 1-methyl-4-phenyl-1,2,3,6-tetrahydropyridine vervet model of parkinsonism. J Neurosci 20: 8559–8571. 11069964 10.1523/JNEUROSCI.20-22-08559.2000 PMC6773163

[14] Moran A, Stein E, Tischler H, Bar-Gad I (2012) Decoupling neuronal oscillations during subthalamic nucleus stimulation in the parkinsonian primate. Neurobiol Dis 45: 583–590. 10.1016/j.nbd.2011.09.016 22001603

[15] Reynolds JH, Chelazzi L (2004) Attentional modulation of visual processing. Annu Rev Neurosci 27: 611–647. 15217345 10.1146/annurev.neuro.26.041002.131039

[16] Mizuseki K, Buzsaki G (2013) Preconfigured, skewed distribution of firing rates in the hippocampus and entorhinal cortex. Cell Rep 4: 1010–1021. 10.1016/j.celrep.2013.07.039 23994479 PMC3804159

[17] Boraud T, Bezard E, Guehl D, Bioulac B, Gross C (1998) Effects of L-DOPA on neuronal activity of the globus pallidus externalis (GPe) and globus pallidus internalis (GPi) in the MPTP-treated monkey. Brain Res 787: 157–160. 9518590 10.1016/s0006-8993(97)01563-1

[18] Drouot X, Oshino S, Jarraya B, Besret L, Kishima H, et al (2004) Functional recovery in a primate model of Parkinson’s disease following motor cortex stimulation. Neuron 44: 769–778. 15572109 10.1016/j.neuron.2004.11.023

[19] Heimer G, Bar-Gad I, Goldberg JA, Bergman H (2002) Dopamine replacement therapy reverses abnormal synchronization of pallidal neurons in the 1-methyl-4-phenyl-1,2,3,6-tetrahydropyridine primate model of parkinsonism. J Neurosci 22: 7850–7855. 12223537 10.1523/JNEUROSCI.22-18-07850.2002 PMC6758069

[20] Moran A, Bergman H, Israel Z, Bar-Gad I (2008) Subthalamic nucleus functional organization revealed by parkinsonian neuronal oscillations and synchrony. Brain 131: 3395–3409. 10.1093/brain/awn270 18986993

[21] Konig P (1994) A method for the quantification of synchrony and oscillatory properties of neuronal activity. J Neurosci Methods 54: 31–37. 7815817 10.1016/0165-0270(94)90157-0

[22] Bair W, O’Keefe LP (1998) The influence of fixational eye movements on the response of neurons in area MT of the macaque. Vis Neurosci 15: 779–786. 9682878 10.1017/s0952523898154160

[23] Softky WR, Koch C (1993) The highly irregular firing of cortical cells is inconsistent with temporal integration of random EPSPs. J Neurosci 13: 334–350. 8423479 10.1523/JNEUROSCI.13-01-00334.1993 PMC6576320

[24] Bair W, Koch C, Newsome W, Britten K (1994) Power spectrum analysis of bursting cells in area MT in the behaving monkey. J Neurosci 14: 2870–2892. 8182445 10.1523/JNEUROSCI.14-05-02870.1994 PMC6577471

[25] Rivlin-Etzion M, Ritov Y, Heimer G, Bergman H, Bar-Gad I (2006) Local shuffling of spike trains boosts the accuracy of spike train spectral analysis. J Neurophysiol 95: 3245–3256. 16407432 10.1152/jn.00055.2005

[26] Erez Y, Czitron H, McCairn K, Belelovsky K, Bar-Gad I (2009) Short-term depression of synaptic transmission during stimulation in the globus pallidus of 1-methyl-4-phenyl-1,2,3,6-tetrahydropyridine-treated primates. J Neurosci 29: 7797–7802. 10.1523/JNEUROSCI.0401-09.2009 19535591 PMC6665635

[27] Welch P (1967) The use of fast Fourier transform for the estimation of power spectra: A method based on time averaging over short, modified periodograms. IEEE Trans Audio Electroacoust 15: 70–73.

[28] Pinhasi Y, Lurie Y (2002) Generalized theory and simulation of spontaneous and super-radiant emissions in electron devices and free-electron lasers. Phys Rev E 65: 1–8. 11863669 10.1103/PhysRevE.65.026501

