# Supplementary material for: Quantifying Spike Train Oscillations: Biases, Distortions and Solutions
Source: PLoS Comput Biol. 2015 Apr 24;11(4):e1004252. doi: 10.1371/journal.pcbi.1004252 (PMC4409360; doi:10.1371/journal.pcbi.1004252)

**Supplementary material 1**

The power spectrum may be defined as the Fourier transform of the auto-correlation function (Wiener-Khinchin theorem), and thus the firing rate induced bias that is illustrated by the power spectrum of the spike train (Fig. S1A-C), could be visualized by the corresponding auto-correlation function of the spike trains (Fig. S1D-F). As the firing rate increases – the oscillatory nature of the spike train is more evident in the auto-correlation function, while much like the power spectrum, the oscillation cannot readily be seen during low rate activity. The SNR of the peak in the auto-correlation of these simulated neurons varies linearly as a function of the base firing rate of the neuron (Fig. S1G). The oscillation frequency cannot typically be assessed using the first-order ISI histogram, regardless the firing rate (Fig S1H-J), as the oscillation relies on ISIs of higher order.


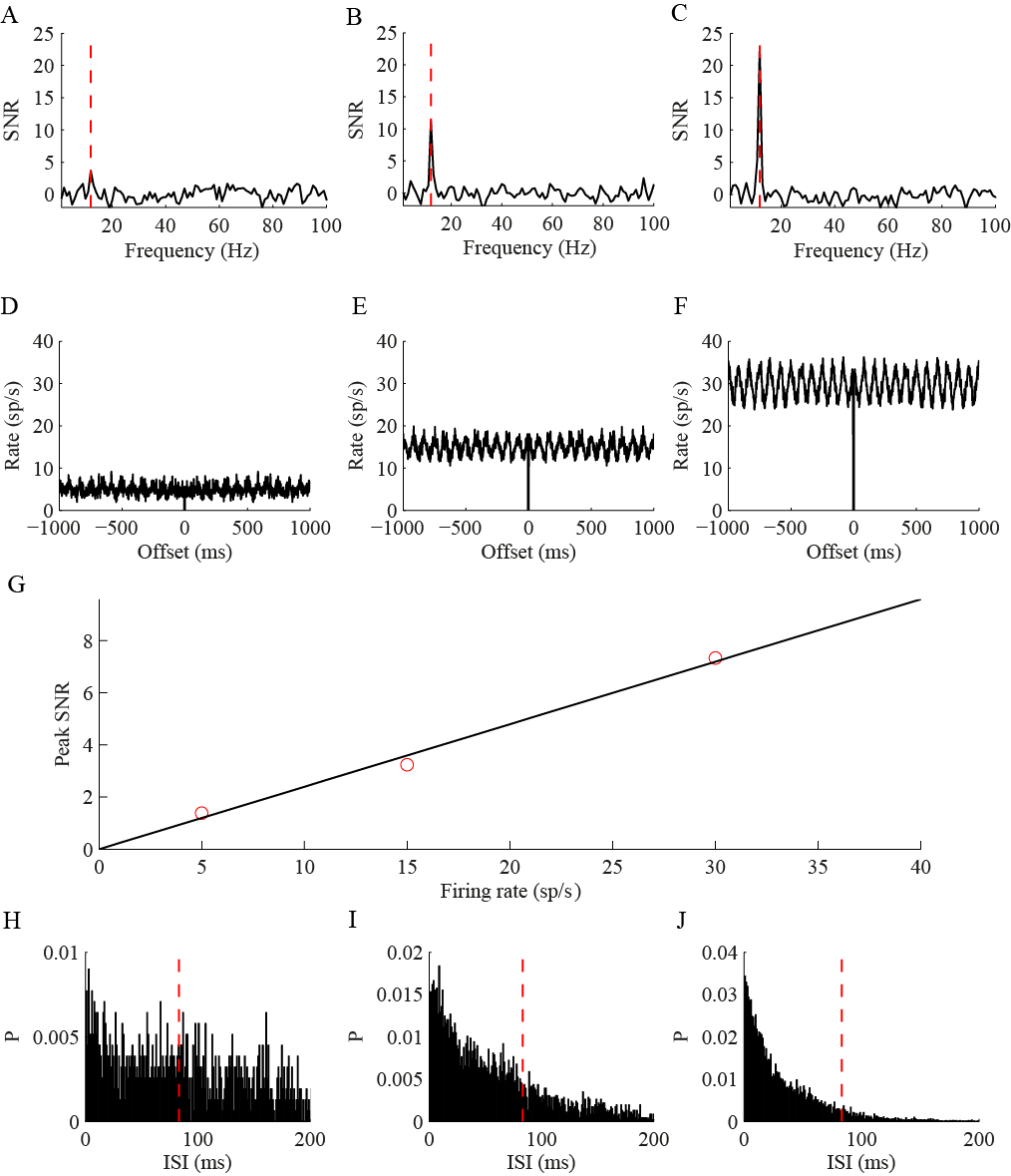

Supplement: S1 Fig — Normalized power spectrum of 5 minutes simulated spike train generated from an oscillatory rate function (f 0 = 12 Hz, m = 0.5), when the base rate is (A) 5 sp/s, (B) 15 sp/s, (C) 30 sp/s. (D-F) The auto-correlation function of the same spike trains as in A-C. (G) The SNR of the peak in the auto-correlation as a function of the mean firing rates. The SNR is defined as the number of STDs from the mean of the auto-correlation function to the first peak near the zero point. The circles indicate the SNR of the peak shown in examples D-F, and the solid line indicates the fitted linear function. (H-J) The first order ISI histograms of the same spike trains shown in A-C. The dashed vertical lines indicate the 1/f 0 ISI. (DOCX) [file pcbi.1004252.s001.docx]
